# Elizabethkingia meningoseptica Infection in COVID-19 Patients

**DOI:** 10.7759/cureus.30337

**Published:** 2022-10-15

**Authors:** Mas Chaponda, Adila Shaukat, Mohammad Wajeh Dulli, Stephanie Sioufi, Walid Al Wali

**Affiliations:** 1 Infectious Diseases Department, Hamad Medical Corporation, Doha, QAT; 2 Internal Medicine Department, Hamad Medical Corporation, Doha, QAT; 3 Microbiology, Hamad Medical Corporation, Doha, QAT

**Keywords:** prognosis, polymicrobial bacteremia, healthcare associated infections, elizabethkingia meningoseptica, bacteremia, antibiotic resistance, covid-19

## Abstract

Bacterial and fungal coinfections including the emergence of antimicrobial resistance are well-recognized in the era of coronavirus disease 2019 (COVID-19) infections. We present three cases of *Elizabethkingia meningoseptica *(EM), superinfections in COVID-19 patients admitted between the period of April 2021 and May 2021. All cases were intubated; had central venous catheters, had received prior antibiotics and antivirals as well as dexamethasone as part of severe COVID-19 management. Only one patient received anakinra. EM isolates were resistant to most available antibiotics and patients infected with it had poor treatment outcomes.

## Introduction

Superinfection is defined as “an infection following a previous infection especially when caused by microorganisms that are resistant or have become resistant to the antibiotics used earlier”, while a co-infection is one occurring concurrently with the initial infection, the difference being purely temporal [1]. During the coronavirus disease 2019 (COVID-19) pandemic more than 70% of patients may receive antibiotics, but <10% experience coinfections [2]. There are numerous publications describing varying ranges of co-infection and superinfection in patients with COVID-19 [2]. These include respiratory viruses, bacteria, mycobacteria, and fungi [1].

An unusual superinfection is *Elizabethkingia meningoseptica* (EM), previously known as *Chryseobacterium meningosepticum* [3]. It is a non-fastidious oxidase-positive, non-glucose fermenting, rod-shaped gram-negative bacterium widely distributed in nature (in water, plants, and soil). It is an uncommon pathogen causing neonatal meningitis, pneumonia, bacteremia, sepsis, soft-tissue infections, and other infections, primarily in immunocompromised patients [3]. Although the mode of transmission is not well known, various reports have suggested a possible association with contaminated water; it is usually acquired in the hospital and hence by definition causes healthcare-associated infections. It is most likely to be associated with the presence of invasive equipment such as intravascular catheters, endotracheal tubes and prosthetic devices, treatment with long-term broad-spectrum antibiotics, long periods of hospitalization and hospital outbreaks [4,5].

EM is inherently resistant to several classes of important antibiotics such as beta-lactams, aminoglycosides, carbapenems, clindamycin, tetracyclines, chloramphenicol, and erythromycin, which are the most common antibiotics used to treat gram-negative nosocomial infections [6]. Infection with this organism requires close collaboration between clinicians and microbiologists to manage, as no interpretive minimum inhibitory concentration (MIC) breakpoints of antibiotics against this organism have been validated to date [6]. EM infections are usually associated with poor clinical outcomes and prognosis [7].

We present three retrospective cases of EM superinfections in COVID-19 patients. All patients were managed at Al Wakra Hospital, Hamad Medical Corporation, Qatar, when it was designated as a COVID-19 facility between April 2021 and May 2021. Hamad Medical Corporation Institutional Review Board issued approval MRC-04-22-026. Microbiology data were obtained from the laboratory information system. Patient demographics, clinical course, imaging, laboratory results, administered antibiotics per days of therapy (DOT), and disposition (admitted, discharged, deceased) were obtained from the electronic medical records (Cerner system). All cases were reviewed by an infectious diseases consultant to determine the presence of clinical superinfection and identification of the primary source. As the case notes were retrospectively reviewed, informed consent was not required according to the local institutional guidelines.

## Case presentation

Case 1

An 80-year-old Qatari woman with known chronic obstructive pulmonary disease (COPD) on home oxygen (ex-smoker of hookah for 40 years, stopped three years prior to admission), BMI of 29, hypothyroidism and hypertension, non-vaccinated for COVID-19, presented with shortness of breath and generalized body pain. She was hypoxic: Oxygen saturation (SpO2) of 86% on room air, respiration rate (RR) 32/min, temperature 36.8^o^C, blood pressure (BP) 106/62, pulse 77 beats/min. Her baseline saturation was 92% at home. Severe acute respiratory syndrome coronavirus 2 (SARS-CoV-2) polymerase chain reaction (PCR) was done and was positive with a cycle threshold (CT) value of 24.4. Her chest X-Ray revealed bilateral patchy opacification. She was diagnosed with severe COVID-19 pneumonia according to local guidance (Adolescent or adult with fever or suspected respiratory infection, plus one of, respiratory rate > 30 breaths/min or severe respiratory distress, or SpO2 < 90% on room air). Supplemental oxygen was given through a nasal cannula, three days later it was escalated to non-invasive ventilation (NIV) 15L, 70% fraction of inspired oxygen (FIO2). COVID-19 pneumonia was treated as per protocol guidelines initially with favipravir then changed when she deteriorated on day three to remdesivir for five days and IV dexamethasone for 10 days. She did not receive anakinra or tocilizumab. On day 4, she continued desaturating, was severely tachypneic and was ultimately intubated in ICU. Due to prolonged intubation and difficulty weaning off oxygen, the patient had a tracheostomy four weeks later. Her hospital course was complicated by ventilator-associated pneumonia (VAP), urinary tract infection (UTI) with *Enterococcus faecalis* and forearm cellulitis which were appropriately treated. During her third week, her C-reactive protein (CRP) increased from 23 to 160 mg/L and blood cultures grew EM. Her central lines were removed and cardiac echography did not show any vegetations. Due to persistence of EM in blood cultures for seven days, she was treated for three weeks with gentamicin and cotrimoxazole guided by sensitivities; levofloxacin was later added. Her endotracheal aspirate also grew *Aspergillus terreus*, *Aspergillus niger*, *Aspergillus nidulans* and *Candida albicans*, for which she completed three weeks of anidulafungin antifungal treatment.

She developed septic shock twice which required vasopressors. She became oliguric with a urine output of 0.4 mL/kg/hour for six hours. Overall, she received multiple courses of antibiotics: piperacillin/tazobactam, meropenem, amoxicillin clavulanate, gentamicin, levofloxacin and cotrimoxazole. She died of septic shock two months after admission.

Case 2

A 49-year-old Indian man, non-smoker with a past medical history of hypertension, type 2 diabetes (T2DM) and chronic kidney disease (CKD) stage 3a, BMI 26.67, non-vaccinated for COVID-19, presented with a four-day history of fever, cough and shortness of breath. He was hypoxic, with an admission SpO2 of 55% on room air, temperature 39^o^C, pulse 132 beats per minute, RR 40/min, BP 149/90. SARS-CoV-2 PCR was positive with a CT value of 17.7 (average CT<30 positive). He was diagnosed with severe COVID-19 pneumonia (Adolescent or adult with fever or suspected respiratory infection, plus one of, respiratory rate > 30 breaths/min or severe respiratory distress, or SpO2 < 90% on room air). He was intubated and admitted to the ICU. His chest X-Ray showed bilateral patchy opacifications. He was initially treated with piperacillin/tazobactam, azithromycin and remdesivir for five days and intravenous dexamethasone for 10 days in accordance with the national COVID-19 treatment protocol. He never received anakinra or tocilizumab. His ICU stay was complicated by failed extubation, a right-sided pneumothorax and subsequent cardiac arrest requiring CPR. A tracheostomy was placed one month after admission. During his ICU course, he was treated for extended-spectrum beta-lactamase (ESBL)-producing *Klebsiella pneumonia* bacteremia, *Serratia marcescens* VAP and bacteremia. Guided by sensitivities, he was initially given meropenem for the *Klebsiella* and intravenous trimethoprim/sulfamethoxazole for the *Serratia*, which was then switched to tigecycline due to acute kidney injury. Three weeks later, he still had EM in his tracheal aspirate which was treated with levofloxacin. His admission was further complicated by *Candida auris* colonization which was treated with topical terbinafine or nystatin in line with local protocol and observation of strict infection prevention and control (IPC) protocols. He died three months after admission from a sudden cardiac arrest.

Case 3

A 56-year-old obese (BMI of 35) Ethiopian woman, non-smoker, non-vaccinated for COVID-19, with a history of asthma on treatment with no other comorbidities presented with fever, cough and shortness of breath. She was hypoxic with her initial SpO2 of 95%r with 8 litres oxygen via a simple mask, RR 25/min, pulse 110 beats/min, BP 176/80. Her SARS-CoV-2 PCR was positive with a CT value of 33.5. She was diagnosed with severe COVID-19 pneumonia (Adolescent or adult with fever or suspected respiratory infection, plus one of, respiratory rate > 30 breaths/min or severe respiratory distress, or SpO2 < 90% on room air). As she deteriorated (SpO2 75%) on continuous positive airway pressure (CPAP), the patient was transferred to ICU and intubated on day 3. She was treated with 10 days IV dexamethasone and remdesivir for five days. Her interleukin 6 level was 49 pg/mL (reference range ≤ 7 pg/mL) on admission. She received anakinra for seven days as per national COVID-19 protocol for severe pneumonia. She failed to wean off ventilatory support and went on to have an elective tracheostomy at five weeks from admission. During her ICU course she had episodes of sepsis which were empirically treated with piperacillin/tazobactam, teicoplanin, amikacin and later meropenem. Anidulafungin was initiated for *Candida galabrate* detected in urine cultures. One month into her ICU admission, she became febrile, tachycardic and hypotensive. Her tracheal aspirate grew EM, which was treated with levofloxacin and gentamicin as per sensitivity results. She also had concurrent bacteremia with *Bacteroides thetaiotaomicron*, for which she received piperacillin/tazobactam. She was also treated for *Candida parapsilosis* candidemia with anidulafungin and for urosepsis with *Leclercia adecarboxylata* isolated from her urine. In addition, she had episodes of bradycardia followed by a cardiac arrest caused by a mucus plug. She was successfully resuscitated, and subsequently developed post-cardiac arrest myoclonic seizures. MRI was done which was highly suggestive of hypoxic encephalopathy. Five months post-admission, the patient with a Glasgow Coma Scale (GCS) of 3 was transferred to a long-term care facility where patients in a vegetative state are managed. She died nine months after admission.

A summary of the baseline characteristics of all three cases is shown in Table 1 below. Also shown are the antibiotic sensitivities of EM strains for all three cases in Table 2.

**Table 1:** Demographics, Comorbidities, and Clinical Characteristics COPD: chronic obstructive pulmonary disease, HTN: hypertension, DMT2: diabetes mellitus type 2, CKD: chronic kidney disease, SARS-CoV-2: severe acute respiratory syndrome coronavirus 2, EM: Elizabethkingia meningoseptica

	Case 1	Case 2	Case 3
Age	80	49	56
Gender	F	M	F
Co-morbidities	COPD, HTN	HTN, DMT2, CKD	Asthma, Morbid obesity
Time between SARS-CoV-2 and EM isolation	25 days	43 days	47 days
Prior antibiotic use	Yes	Yes	Yes
Prior biologic use	No	No	Yes
Prior dexamethasone use	Yes	Yes	Yes
Pulmonary dysfunction	Yes	Yes	Yes
Renal dysfunction	Yes	Yes	No
Hepatic dysfunction	No	No	No

**Table 2:** Result of antimicrobial susceptibility testing S susceptible, I intermediate, R resistant

Antibiotic	Cases		
	1	2	3
Amikacin Gentamicin	S	I	S
Ceftazidime	R	R	R
Ciprofloxacin	R	R	R
Ceftriaxone	R	R	R
Imipenem	R	R	R
Levofloxacin	S	S	S
Meropenem	R	R	R
Trimethoprim/sulfamethoxazole (Septrin)	S	R	R
Piperacillin/tazobactam (Tazocin)	R	R	R
Colistin	R	R	R
Tetracycline	R	S	R
Moxifloxacin	S	S	S

## Discussion

Several publications have shown that microbiologically confirmed infections were infrequent in patients admitted to hospital with COVID-19 and that co-infection at hospital admission was rare [8]. The cases presented had polymicrobial organisms isolated from the tracheal aspirates and on occasions from their blood culture weeks out from the initial COVID-19 diagnosis. This is not surprising and has been described in ICU patients with prolonged stay [8].

On review of published literature on EM infection [7], it is noted that the cases presented had the following risk factors: all patients were ventilated for more than six weeks, one case had COPD, and another had chronic kidney disease and diabetes mellitus. However, none had had major surgery or a diagnosis of heart failure [7]. Our three cases had pulmonary dysfunction (hypoxia), two cases also had renal dysfunction (acute kidney injury [AKI] and CKD) and none had evidence of hepatic dysfunction (derangement in liver function tests and coagulation). Comparative cases of EM had multiple risk factors for nosocomial infections, such as critical illness, prolonged hospitalization, mechanical ventilation, and immune dysregulation [2]. One case received biologic agents, namely anakinra and tocilizumab, which were used for the treatment of cytokine release syndrome [9]. These agents have been shown to weaken immune responses and may be associated with/predisposing to unusual bacterial infections, especially in patients with pre-existing comorbidities. All patients had at least 10 days of dexamethasone as per COVID-19 treatment protocol for severe pneumonia [10]. Steroids can, of course, also predispose to bacterial coinfection.

The authors recognize that the EM infections reported in our cases might have been secondary to a nosocomial outbreak. However, while there is a temporal association between the cases, there is no co-location as patients were on different floors and areas of the hospital, thus remaining geographically distant. In addition, we follow strict infection control programs including care bundles for central line-associated bloodstream infection (CLABSI) and VAP, isolation precautions, and a hand hygiene program. The case of an outbreak remains nonetheless a possibility to consider.

All our patients had extensive antibiotic exposure in the 30 days preceding positive EM microbiology. The clinical presentation of severe COVID-19 may be indistinguishable from bacterial or fungal sepsis, which is likely driving excess antimicrobial use [7]. In addition, all three patients developed, at some point, bacteremia and polymicrobial organisms were isolated from their tracheal aspirates including *Serratia marcescens* and *Enterococcus faecalis*, which were treated with broad-spectrum antibiotics. Because of its resistance to carbapenems, the increasing use of this class of antibiotic for severe nosocomial infections caused by extended-spectrum β-lactamase-producing gram-negative bacteria, such as *Pseudomonas* and *Acinetobacter*, might contribute to the emergence of EM infection, particularly in the ICU [1].

The choice of optimal antibiotic agents for treating EM infection is difficult because of the unpredictability and breadth of antimicrobial resistance of this organism, such as β-lactam agents, aminoglycosides and carbapenems [11,12]. All isolates were susceptible to levofloxacin whilst trimethoprim/sulfamethoxazole and gentamicin were only effective to a certain degree. All EM isolates showed resistance to ceftriaxone, ceftazidime, piperacillin/tazobactam, ciprofloxacin and meropenem. The most common antibiotics used to treat EM bacteremia after the results of drug susceptibility tests were available were trimethoprim/sulfamethoxazole.

Notably, in all cases, there was persistence/colonization of EM in the tracheal aspirates which over time developed resistance to trimethoprim/sulfamethoxazole. The patients were then treated with combination therapy consisting of levofloxacin, gentamicin or tigecycline.

The median antibiotic DOT was 10 days (IQR, 7-21) with more than three antibiotic classes received, which compares with other series in the literature [13]. Two patients with EM bacteremia died and the third was left with considerable disability. This is in contrast to the low overall COVID-19 fatality rate in Qatar placed at 0.14% in July 2020 [14].

There has been one published case report of COVID-19 infection with subsequent EM requiring intubation and mechanical ventilation. Furthermore, the patient had comorbidities (hypertension, cerebrovascular disease) and developed an acute kidney injury over the course of his hospital stay. These similarities are indeed reflected in the case series presented here, emphasizing the risk associated with multimorbid patients admitted and treated for severe COVID-19 pneumonia. However, our case series highlights the incidence of multiple concomitant infections - including fungi - in these patients with EM bacteremia, as opposed to one instance of hospital-acquired pneumonia in the previously published case report [15].

## Conclusions

*E. meningoseptica* is an alert organism from the infection prevention control perspective which we have encountered in patients with recent severe COVID-19 pneumonia and prolonged ICU admission. It intrinsically has a high degree of resistance to most of the routinely used broad-spectrum antibiotics. Effective rapport between the clinician and the microbiologist is crucial in the optimal management of these patients.
